# Gender-specific association of decreased estimated glomerular filtration rate and left vertical geometry in the general population from rural Northeast China

**DOI:** 10.1186/s12872-016-0459-0

**Published:** 2017-01-13

**Authors:** Dongxue Dai, Ye Chang, Yintao Chen, Shasha Yu, Xiaofan Guo, Yingxian Sun

**Affiliations:** Department of Cardiology, the First Hospital of China Medical University, 155 Nanjing North Street, Heping District, Shenyang, 110001 People’s Republic of China

**Keywords:** Left ventricular geometry, Estimated glomerular filtration rate, Gender difference, General population

## Abstract

**Background:**

Left ventricular hypertrophy (LVH) is common and associated with cardiovascular outcomes among patients with known chronic kidney disease (CKD). However, the link between decreased estimated glomerular filtration rate (eGFR) and left ventricular (LV) geometry remains poorly explored in general population. In this study, we examined the gender-specific association between eGFR and LVH in the general population from rural Northeast China.

**Methods:**

This survey was conducted from July 2012 to August 2013. A total of 10907 participants (5,013 men and 5,894 women) from the rural Northeast China were randomly selected and examined. LV mass index (LVMI) was used to define LVH (LVMI > 46.7 g/m^2.7^ in women; > 49.2 g/m^2.7^ in men). LV geometry was defined as normal, or with concentric remodeling, eccentric or concentric hypertrophy, according to relative wall thickness (RWT) and LVMI. Mildly decreased eGFR was defined as eGFR ≥ 60 and < 90 ml/min/1.73 m^2^, and moderate-severely decreased eGFR was defined as eGFR < 60 ml/min/1.73 m^2^.

**Results:**

As eGFR decreased, LVH showed a gradual increase in the entire study population. Multivariate analysis revealed a gender-specific relationship between eGFR and LV geometry. Only in men, mildly decreased eGFR was associated with concentric remodeling [odds ratio (OR): =1.58; 95% CI: 1.14–2.20; *P* < 0.01] and concentric LVH *OR* = 1.63; 95% CI: 1.15–2.31; *P* < 0.01). And only in men, moderate-severely decreased eGFR was a risk factor for concentric LVH (*OR* = 4.56; 95% CI: 2.14–9.73; *P* < 0.001) after adjusting for confounding factors.

**Conclusions:**

These findings suggested that decreased eGFR was a risk factor for LV geometry in men, and a gender-specific difference should be taken into account in clinical practice.

## Background

The dramatically increasing prevalence of chronic kidney disease (CKD) is a great challenge to public health globally and also constitutes serious economic burden [1]. Reduced estimated glomerular filtration rate (eGFR), one important marker of renal dysfunction, is a major risk factor for cardiovascular disease (CVD) and has a significant influence on the outcomes of the patients with CVD [2]. Previous study has indicated that even a mild decrease in eGFR could increase the risk of cardiovascular morbidity and all-cause mortality [3]. Consensus is reached that the relationship between renal and cardiac disease is close and bidirection. Reduced eGFR is often associated volume retention and increased preload, which could trigger several biological signal cascades leading to cardiac hypertrophy [4]. On the other hand, preexisting cardiac disease can accelerate renal damage and result in rapid deterioration of eGFR [5].

Left ventricular hypertrophy (LVH), one type of cardiac disease, emerges as an adaptive response to pressure or volume overload, appears to be a typical adverse cardiac remodeling that occurs in CKD patients with decreased eGFR [6]. Abnormal cardiac structure and function are increased the risk of cardiovascular morbidity and mortality [7]. Most studies pay much attention to LVH because it is an independent risk factor of myocardial infarction, heart failure, arrhythmia and other cardiovascular death [8, 9].

Various degree of decreased eGFR have significant effects on the development of LVH [3, 10–12], and the available evidence showed that severely decreased eGFR was strongly associated with increased left ventricular mass (LVM) and increased prevalence of LVH [13]. Accumulating evidence indicates that the prevalence of LVH is inversely parallel to the value of eGFR [13, 14]. However, few studies have investigated the effects of gender on the correlation of eGFR and LVH. Furthermore, LVH could be furthermore classified into concentric remodeling, concentric hypertrophy, and eccentric hypertrophy based on left ventricular mass index (LVMI) and relative wall thickness (RWT) [15]. Data on reduced eGFR and LV geometry are scarce.

We performed this study with the aims: 1) improving LVH prevention by identifying its risk factors; 2) clarifying whether prevalence of LVH is significantly correlated with reduced eGFR; and 3) identifying gender-specific association in a general population in rural Northeast China.

## Methods

### Approvals

The study was approved by the Ethics Committee of China Medical University (Shenyang, China). All procedures were performed under the relevant ethical standards. Written consents were obtained from all participants after they had been informed of the objectives, benefits, medical items and confidentiality agreement regarding their personal information. For participants who were illiterate, we obtained written informed consents from their proxies.

### Study population

From January 2012 to August 2013, a representative sample of individuals in rural areas of Liaoning Province was recruited to present the prevalence, incidence and natural history of cardiovascular risk factors in rural areas of Liaoning Province. The study was a multi-stage, stratified, random-cluster sampling scheme. Firstly, three counties (Dawa, Zhangwu and Liaoyang County) were randomly selected from rural areas of Liaoning province. Then, one town was randomly selected from each three counties. At last, a total of 26 rural villages from the three towns were randomly selected. Participants with pregnant or malignant tumors or mental disorders were excluded from the study. A total of 14,016 residents aged ≥ 35 years from each village were invited to participant the study and 11,956 participants (i.e. response rate of 85.3%) agreed and completed the study. In this report, we finally enrolled 10907 participants (5,013 men and 5,894 women) to examine the association between decreased eGFR and LV structural alterations in the Chinese Northeast population.

### Echocardiography measurements

The echocardiograms were obtained using a commercially available Doppler echocardiograph (Vivid, GE Healthcare, United States), with a 3.0-MHz transducer. The LV dimension including end-diastolic LV internal diameter (LVEDD), ventricular septal thickness (IVST) and posterior LV wall thickness (PWT) were ascertained as guidance with the recommendations of the American Society of Echocardiography [16]. LVM was calculated according to the formula: LVM (g) = 0.81 × (1.04 × [LVEDD + IVST + PWT]) ^3^ - (LVEDD) ^3^ + 0.06 [17]. LVH was defined as the LVM/height^2.7^ > 46.7 g/m^2.7^ in women and > 49.2 g/m^2.7^ in men [18]. The relative wall thickness (RWT) = 2 × PWT/LVEDD at end-diastole and considered increased if > 0.43 [15]. LV geometry was assessed from the LVM/height^2.7^ combined with the RWT [15, 17]. Concentric remodeling was defined by normal LVM/height^2.7^ and increased RWT, eccentric hypertrophy by increased LVM/height^2.7^ and normal RWT, and concentric LV hypertrophy by increased LVM/height^2.7^ and increased RWT.

### Covariate measurements

Those participants who met with the inclusion criteria completed a comprehensive questionnaire including questions about age, education, family income, physical activity, consumption of alcohol and cigarette in a face-to-face interview. Before the survey was performed, we invited all eligible investigators to attend an organized training session and a strict test was evaluated to screen out qualified investigators. During data collection, our inspectors would support further instructions.

Blood pressure (BP) was measured with a standardized automatic electronic sphygmomanometer (HEM-907; Omron) in accordance with the British Hypertension Society protocol [19]. The participants were advised to avoid caffeinated beverages and exercise for at least 30 min before the measurement. During the measurement, the participants were seated with their arms supported at the level of the heart and BP was taken three times at 2-min intervals after at least 5 min of rest. The mean of three BP measurements was calculated and used in all analyses. The participants were informed of wearing light-weight clothing and without shoes to obtain anthropometric data on weight and height, which were measured respectively to the nearest 0.1 kg and 0.1 cm. Body mass index (BMI) was calculated as the weight in kilograms divided by the square root of the height in meters.

Fasting blood samples were collected in the morning after at least 12 h of fasting for all participants. Blood samples were obtained from an antecubital vein and collected in vacutainer tubes containing EDTA. Blood chemical analyses were performed at a central, certified laboratory. Serum creatinine (SCr) was measured enzymatically on an autoanalyzer. GFR was estimated using the equation originating from the CKD Epidemiology Collaboration (CKD-EPI) equation [20], which is more appropriate than the Modification of Diet in Renal Disease (MDRD) Study group equation [21]. From this population, we enrolled participants with normal eGFR defined as eGFR ≥ 90 ml/min/1.73 m^2^. Mildly decreased eGFR was defined as 60–90 ml/min/1.73 m^2^, and moderate-severely decreased eGFR defined as eGFR < 60 ml/min/1.73 m^2^.

In addition, hemoglobin (Hb), fasting plasma glucose (FPG), total cholesterol (TC), low-density lipoprotein cholesterol (LDL-C), high-density lipoprotein cholesterol (HDL-C), triglycerides (TG), and other routine blood biochemical indexes were analyzed enzymatically using an autoanalyzer. All laboratory equipments were calibrated, and blinded duplicate samples were used for these analyses.

### Definitions

According to JNC-7 report [22], hypertension was defined as systolic blood pressure (SBP) ≥ 140 mmHg and/or diastolic blood pressure (DBP) ≥ 90 mmHg and/or use of antihypertensive medications. Dyslipidemia was defined according to the National Cholesterol Education Program-Third Adult Treatment Panel (ATP III) criteria [23]. Hyperuricemia was defined as serum uric acid > 375 umol/L in women and > 416 umol/L in men according to guidelines [24].

### Statistical analysis

Descriptive statistics were calculated for all the variables, including continuous variables (reported as mean values and standard deviations) and categorical variables (reported as numbers and percentages). Comparisons were performed using t tests, χ^2^ tests, and one-way ANOVAs. Multivariate logistic regression analyses were used to identify the association between eGFR and LV geometry in men and women respectively, and odds ratios (ORs) and corresponding 95% confidence intervals (CIs) were calculated. All the statistical analyses were performed using SPSS version 22.0 software, and *p* values < 0.05 were considered to be statistically significant.

## Results

### Baseline clinical characteristics

The baseline characteristics of the 10,907 participants with a mean age of 53.9 ± 10.5 years are summarized in Table 1. The level of eGFR for men was higher compared with women (93.97 ± 15.38 vs. 91.69 ± 16.26; *P* < 0.001). Men had higher mean BP and lower TC than women. The level of hemoglobin was significantly lower in women than in men.

**Table 1:** Baseline characteristics of study population by gender

Variables	All	Men	Women	*P*-value
	(*N* = 10907)	(*N* = 5013)	(*N* = 5894)	
Age (years)	53.9 ± 10.5	54.4 ± 10.8	53.4 ± 10.3	<0.001
Smokers (%)	3833	2872 (57.3)	961 (16.3)	<0.001
Drinkers (%)	2444	2278 (45.4)	166 (2.8)	<0.001
SBP (mmHg)	141.55 ± 23.32	143.42 ± 22.49	139.96 ± 23.89	<0.001
DBP (mmHg)	81.98 ± 11.74	83.69 ± 11.78	80.52 ± 11.50	<0.001
TC (mmol/L)	5.24 ± 1.09	5.18 ± 1.04	5.30 ± 1.12	<0.001
TG (mmol/L)	1.63 ± 1.48	1.65 ± 1.63	1.62 ± 1.35	0.267
LDL-C (mmol/L)	2.93 ± 0.82	2.88 ± 0.79	2.97 ± 0.84	<0.001
HDL-C (mmol/L)	1.40 ± 0.38	1.40 ± 0.42	1.41 ± 0.34	0.675
Uric acid (mmol/L)	291.86 ± 85.08	334.41 ± 83.55	67.89 ± 0.88	<0.001
FPG (mmol/L)	5.91 ± 1.63	5.95 ± 1.65	5.87 ± 1.61	0.011
eGFR (ml/min/1.73 m^2^)	93.97 ± 15.38	93.97 ± 15.38	91.69 ± 16.26	<0.001
Hemoglobin (g/L)	138.64 ± 18.70	148.64 ± 18.18	130.13 ± 14.43	<0.001
ECG indices				
IVST (cm)	0.88 ± 0.24	0.92 ± 0.27	0.85 ± 0.22	<0.001
LVEDD (cm)	4.71 ± 0.43	4.90 ± 0.41	4.55 ± 0.38	<0.001
PWT (cm)	0.87 ± 0.27	0.98 ± 0.28	0.84 ± 0.25	<0.001
RWT	0.37 ± 0.09	0.37 ± 0.09	0.37 ± 0.09	0.104
LVM (g)	143.11 ± 98.28	159.69 ± 107.28	129.00 ± 87.48	<0.001
LVM/H^2.7^ (g/h^2.7^)	40.79 ± 27.42	41.45 ± 27.31	40.22 ± 27.49	0.020

*P* values for comparisons were performed with t tests for continuous variables and χ^2^ test for categorical variables. *SBP* systolic blood pressure, *DBP* diastolic blood pressure, *TC* triglyceride, *TG* total cholesterol, *HDL*-*C* high-density lipoprotein, *LDL*-*C* low-density lipoprotein, *FPG* fasting plasma glucose, *eGFR* estimated glomerular filtration rate, *ECG* echocardiogram, *IVST* interventricular septum thickness, *LVEDD* left ventricular end-diastolic dimension, *PWT* posterior wall thickness, *RWT* relative wall thickness

The mean value of IVST, LVEDD, PWT, LVM and LV mass indexed for height^2.7^ in women were 0.85 ± 0.22, 4.55 ± 0.38, 0.84 ± 0.25 cm, 129.00 ± 87.48 g and 40.22 ± 27.49 g/h^2.7^, respectively, which were all significantly lower than those in men.

### Baseline characteristics of study population according to eGFR

As shown in Table 2, participants with decreased eGFR tended to be older and female. SBP, DBP, FPG, LDL-C, TC, TG, and uric acid were significantly higher in moderate-severely decreased eGFR group, but hemoglobin and HDL-C showed the opposite trend. Additionally, subjects with normal eGFR were more likely to be smokers and drinkers. Moreover, participants with decreased eGFR had a higher value of IVST, LVEDD, PWT, LVM and LV mass indexed for height^2.7^.

**Table 2:** Baseline characteristics of study population by eGFR

Variables	eGFR ≥ 90 (ml/min/1.73 m^2^)	90 > eGFR ≥ 60 (ml/min/1.73 m^2^)	eGFR < 60 (ml/min/1.73 m^2^)	*P*-value
	*N* = 6578	*N* = 4098	*N* = 231	
Age (years)	49.86 ± 8.67	60.00 ± 10.01	68.44 ± 9.48	<0.001
Male (%)	3289 (50.0)	1643 (40.1)	81 (35.1)	<0.001
Smokers (%)	2490 (37.9)	1275 (33.3)	68 (29.4)	<0.001
Drinkers (%)	1773 (27.0)	655 (16.0)	16 (0.7)	<0.001
SBP (mmHg)	139.28 ± 22.47	144.34 ± 23.83	156.63 ± 26.72	<0.001
DBP (mmHg)	81.44 ± 11.54	82.67 ± 11.75	85.00 ± 15.51	<0.001
TC (mmol/L)	5.09 ± 1.03	5.45 ± 1.09	5.82 ± 1.65	<0.001
TG (mmol/L)	1.56 ± 1.55	1.73 ± 1.33	2.13 ± 1.73	<0.001
LDL-C (mmol/L)	2.85 ± 0.79	3.03 ± 0.83	3.27 ± 1.11	<0.001
HDL-C (mmol/L)	1.45 ± 0.40	1.35 ± 0.33	1.30 ± 0.37	<0.001
Uric acid (mmol/L)	297.37 ± 81.70	306.10 ± 82.35	394.83 ± 113.27	<0.001
FPG (mmol/L)	5.82 ± 1.65	6.01 ± 1.52	6.59 ± 2.42	<0.001
Hemoglobin (g/L)	140.03 ± 19.08	136.84 ± 17.80	130.98 ± 18.63	<0.001
ECG indices				
IVST (cm)	0.87 ± 0.19	0.91 ± 0.31	0.97 ± 1.58	<0.001
LVEDD (cm)	4.72 ± 0.41	4.70 ± 0.44	4.77 ± 0.57	0.016
PWT (cm)	0.86 ± 0.24	0.88 ± 0.28	0.96 ± 0.49	<0.001
RWT	0.36 ± 0.11	0.38 ± 0.14	0.41 ± 0.21	<0.001
LVM (g)	143.00 ± 81.87	150.76 ± 119.51	172.48 ± 118.24	<0.001
LVM/H^2.7^ (g/h^2.7^)	39.26 ± 22.73	42.70 ± 33.33	50.36 ± 29.38	<0.001

*P* values for comparisons were performed with ANOVA for continuous variables and χ^2^ test for categorical variables. *ANOVA* analysis of variance, *eGFR* estimated glomerular filtration rate, *SBP* systolic blood pressure, *DBP* diastolic blood pressure, *TC* triglyceride, *TG* total cholesterol, *HDL*-*C* high-density lipoprotein, *LDL*-*C* low-density lipoprotein, *FPG* fasting plasma glucose, *ECG* echocardiogram, *IVST* interventricular septum thickness, *LVEDD* left ventricular end-diastolic dimension, *PWT* posterior wall thickness, *RWT* relative wall thickness

**Fig. 1:**
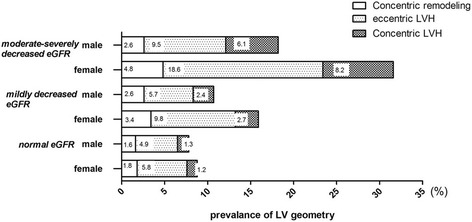
Prevalance of abnormal LV geometry according to different eGFR and gender

Meanwhile, Fig. 1 showed that the prevalance of different cardiac remolding increased with the aggravation of impaired kidney function, especially the concentric hypertrophy.

### Baseline characteristics of study population according to LV geometry

Based on criteria listed in the methods, we divided the study population into 4 groups according to LV geometry: normal LV geometry (*n* = 8610; 78.94%), concentric remodeling (*n* = 485; 4.45%), eccentric hypertrophy (*n* = 1403; 12.86%), and concentric hypertrophy (*n* = 409; 3.74%). The mean age of subjects with normal LV geometry were 52.5 ± 10.1 years, which was significantly lower than that of those who had either eccentric or concentric LVH. The eGFR level gradually decreased in the four groups. Participants with concentric or eccentric hypertrophy had significantly lower levels of HDL-C and significantly higher levels of uric acid and FPG, SBP and DBP, compared to those with normal LV geometry (Table 3).

**Table 3:** Baseline characteristics of study population by cardiac geometry

Variables	Normal geometry	Concentric remodeling	Eccentric LVH	Concentric LVH	*P*-value
	(*N* = 8610)	(*N* = 485)	(*N* = 1403)	(*N* = 409)	
Age (years)	52.5 ± 10.1	57.7 ± 11.1	59.0 ± 10.2	59.8 ± 10.6	<0.001
Male (%)	4590 (53.3)	268 (55.3)	827 (59.0)	209 (51.1)	<0.001
Smokers (%)	3081 (35.8)	166 (34.2)	433 (30.9)	153 (37.4)	0.003
Drinkers (%)	1983 (23.0)	106 (21.9)	257 (18.3)	98 (24.0)	0.001
SBP (mmHg)	137.61 ± 20.75	147.56 ± 26.16	155.93 ± 25.23	167.97 ± 25.74	<0.001
DBP (mmHg)	80.66 ± 10.82	84.49 ± 12.89	86.26 ± 13.22	92.14 ± 14.32	<0.001
TC (mmol/L)	5.19 ± 1.06	5.40 ± 1.13	5.42 ± 1.20	5.63 ± 1.12	<0.001
TG (mmol/L)	1.57 ± 1.45	1.78 ± 1.57	1.87 ± 1.63	1.95 ± 1.34	<0.001
LDL-C (mmol/L)	2.89 ± 0.80	2.99 ± 0.84	3.06 ± 0.88	3.26 ± 0.95	<0.001
HDL-C (mmol/L)	1.41 ± 0.38	1.46 ± 0.47	1.35 ± 0.36	1.37 ± 0.37	<0.001
Uric acid (mmol/L)	287.89 ± 83.11	302.07 ± 92.25	302.70 ± 88.97	326.12 ± 91.74	<0.001
FPG (mmol/L)	5.84 ± 1.54	6.11 ± 1.79	6.17 ± 1.91	6.32 ± 1.97	<0.001
eGFR (ml/min/1.73 m^2^)	94.14 ± 15.49	88.11 ± 14.81	87.92 ± 16.03	85.19 ± 18.57	<0.001
Hemoglobin (g/L)	138.82 ± 19.26	138.64 ± 15.07	136.95 ± 16.23	140.64 ± 18.11	0.001
ECG indices					
IVST (cm)	0.84 ± 0.08	0.97 ± 0.10	1.05 ± 0.56	1.15 ± 0.33	<0.001
LVEDD (cm)	4.67 ± 0.37	4.15 ± 0.35	5.14 ± 0.47	4.73 ± 0.39	<0.001
PWT (cm)	0.82 ± 0.07	0.96 ± 0.09	0.94 ± 0.09	1.39 ± 1.18	<0.001
RWT	0.36 ± 0.04	0.47 ± 0.04	0.39 ± 0.11	0.54 ± 0.30	<0.001
LVM (g)	128.69 ± 25.60	130.85 ± 30.95	199.53 ± 173.05	267.63 ± 330.52	<0.001
LVM/H^2.7^ (g/h^2.7^)	36.11 ± 5.98	37.56 ± 6.82	59.92 ± 45.78	77.52 ± 94.83	<0.001

*P* values for comparisons were performed with ANOVA for continuous variables and χ^2^ test for categorical variables. *ANOVA* analysis of variance, *SBP* systolic blood pressure, *DBP* diastolic blood pressure, *TC* triglyceride, *TG* total cholesterol, *HDL*-*C* high-density lipoprotein, *LDL*-*C* low-density lipoprotein, *FPG* fasting plasma glucose, *eGFR* estimated glomerular filtration rate, *ECG* echocardiogram, *IVST* interventricular septum thickness, *LVEDD* left ventricular end-diastolic dimension, *PWT* posterior wall thickness, *RWT* relative wall thickness

### Difference in the relationship between of eGFR and LVH according to gender

Participants were categorized into three groups on the basis of eGFR level. After adjusting for age, hypertension, hemoglobin, dyslipidemia, hyperuricemia, current smoking and drinking, multivariate logistic regression analysis revealed that the significant association between mildly decreased eGFR and concentric remodeling persisted (*OR* = 1.38; 95% CI: 1.11–1.72, *P*-value < 0.01, Table 4). There was still a significant association between moderate-severely decreased eGFR and concentric LVH (*OR* = 2.48; 95% CI: 1.50–4.08; *P* < 0.001, Table 4). Moreover, moderate-severely decreased eGFR was also an independent risk factor for eccentric LVH in the whole population (*OR* = 1.07; 95% CI: 0.74–1.53; *P* = 0.02, Table 4).

**Table 4:** Multivariate analysis of the relationship between CKD and LV geometry after adjusting for confounding factors

eGFR	Concentric remodeling		Eccentric LVH		Concentric LVH	
	OR (95%CI)	*p* value	OR (95%CI)	*p* value	OR (95%CI)	*p* value
Normal	Reference		Reference		Reference	
Mildly decreased	1.38 (1.11–1.72)	<0.01	0.85 (0.74–0.97)	0.74	1.23 (0.96–1.57)	0.10
Moderate-severely decreased	1.47 (0.82–2.63)	0.20	1.07 (0.74–1.53)	0.02	2.48 (1.50–4.08)	<0.001
Male						
Normal	Reference		Reference		Reference	
Mildly decreased	1.58 (1.14–2.20)	<0.01	0.95 (0.77–1.19)	0.67	1.63 (1.15–2.31)	<0.01
Moderate-severely decreased	1.77 (0.68–4.60)	0.24	1.69 (0.93–3.07)	0.09	4.56 (2.14–9.73)	<0.001
Women						
Normal	Reference		Reference		Reference	
Mildly decreased	1.21 (0.91–1.62)	0.19	0.76 (0.63–0.91)	<0.01	0.93 (0.66–1.30)	0.66
Moderate-severely decreased	1.29 (0.61–2.70)	0.50	0.70 (0.44–1.11)	0.13	1.40 (0.71–2.76)	0.33

Confounding factors include age, hypertension, diabetes mellitus, dyslipidemia, hyperuricemia, current smoking and drinking status

In men, we found mildly decreased eGFR was significantly associated with concentric remodeling (*OR* = 1.58; 95% CI: 1.14–2.20; *P* < 0.01, Table 4). However, the association between decreased eGFR and eccentric LVH in men did not achieve statistical significance after adjusting for confounding factors mentioned above. In addition, both mildly and moderate-severely decreased eGFR had various degrees of impact on concentric LVH in men (*OR* = 1.63; 95% CI: 1.15–2.31; *P* < 0.01 vs *OR* = 4.56; 95% CI: 2.14–9.73; *P* < 0.001, Table 4). Conversely in women, decreased eGFR was not a significant risk factor for the different alteration of LV geometry.

### Prediction of LV geometry according to eGFR

**Fig. 2:**
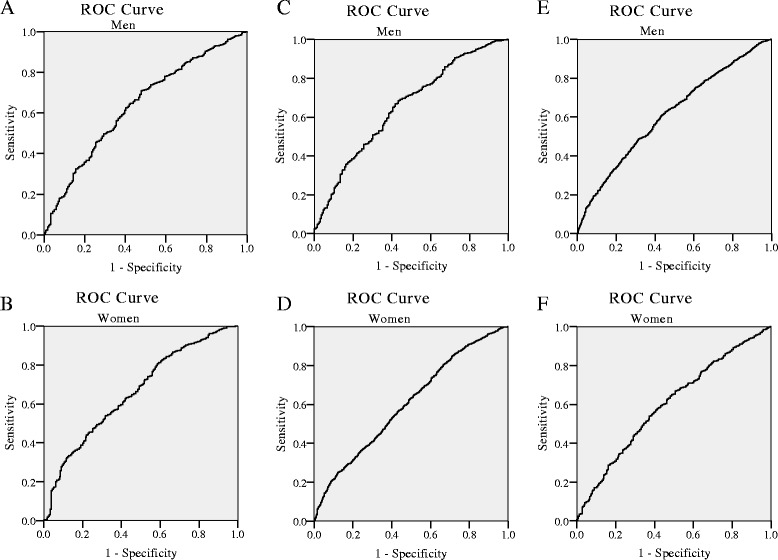
The area under the receiver operating characteristic (ROC) curves of eGFR for the presence of LV geometry in both genders

Figure 2 showed the AUCs (and 95% CIs) of eGFR in the prediction of LV geometry. For concentric remolding, the AUC was 0.64 (95% CIs: 0.60–0.67) for male and 0.60 (95% CIs: 0.57–0.63) for female. For eccentric LVH, the AUC for was 0.61 (95% CIs: 0.60–0.64) for male and 0.60 (95% CIs: 00.58–0.63) for female. For concentric LVH, the AUC was 0.65 (95% CIs: 0.610–0.69) for male and 0.66 (95% CIs: 0.62–0.70) for female.

## Discussion

Echocardiographic studies in Caucasians, African Americans and East Asians, indicate that a decline in renal function is associated with LVH, particularly in patients with both diabetes and hypertension [11, 25, 26]. However, few studies have reported on the association between decreased eGFR and LV geometry. The main finding of our study was that different degrees of decreased eGFR were associated with different types of LV geometry and gender-specific variations existed. In men but not in women, mildly decreased eGFR was significantly associated with concentric remodeling and concentric LVH, and moderate-severely decreased eGFR was an independent risk factor for concentric LVH only in men. These relationships were attenuated by adjusting for confounding factors. To our knowledge, this is the first study to report an association between decreased eGFR and LV geometry in an Asian population and the first one to find a gender-specific difference in the relationship of decreased eGFR and LV geometry.

Consistent with previous studies [11, 13, 26], we found that the prevalence of LVH generally increased in both men and women as eGFR decreased. However, few studies have investigated the influence of eGFR on LV geometry in the general population. Previous study based on children aged 3–18 years found that substantial cardiac remodeling of both concentric and eccentric type was present at young age and early stages of chronic renal insufficiency (GFR 49 ± 19 ml/min/1.73 m^2^) and prevalence of LVH was related to male gender [27]. We found that an association between decreased eGFR and concentric LVH existed in a general population, which was consistent with previous studies reporting the association between CKD with concentric hypertrophy [28, 29]. Ernesto et al. reported that the risk of concentric LVH was increased 2.96-fold in hypertensive patients with an eGFR <30 ml/min/1.73 m^2^ compared to those with an eGFR >60 ml/min/1.73 m^2^, whereas the association of decreased eGFR and eccentric LVH was not significant [30]. The observed increase in aortic stiffness might explain the risk of concentric LV geometry in the study participants with decreased eGFR. In both clinical studies and animal models, aortic stiffness exposed the left ventricle to increased afterload, which was linked with concentric LVH [31, 32].

Differences in the association of renal dysfunction and LV geometry in men and women have not been extensively evaluated. In this population, decreased eGFR was associated with concentric remolding and hypertrophy only in men. Matteucci et al. reported that in children with chronic renal insufficiency, the risk of LVH was four-fold greater in boys than in girls [27] and Meisinger et al. found that men with CKD had higher concentrations of urinary albumin and greater cardiovascular risk than women [33]. Concentric LVH is believed to result primarily from hypertension and increased afterload [34]. In the present study, men had significantly higher values of BP and FPG, so it was quite possible for them to have greater aortic stiffness and lower total arterial compliance. Consequently, men might be more vulnerable to the deleterious effects of early arterial load on diastolic function and ventricular-arterial interaction, which could be a reason for the gender-specific results. In addition, the gender difference in the range of eGFR that promotes CVD might be explained in part by estrogen effects. Some studies found that estrogen had protective effects on cardiac structure. Li et al. reported that the estrogen could resist adverse cardiac remodeling in a rat transverse aortic constriction or sham surgery model by inhibiting release of chymase [35]. Other studies also confirmed that in a rat transverse aortic constriction or sham surgery model [36].

The possible causes of LV geometry have been reviewed recently, and although several studies point to the involvement of age, anemia [37], inflammation [38], oxidative stress [39] and disordered mineral metabolism [40], the effects of those variables remain unclear. Gender-specific difference in eGFR-associated LVH should be fully considered in studies of treatment and prevention strategies.

### Limitations

There are some limitations of our study: (1) the present study was a cross-sectional design, which restricted the establishment of the cause and effect relationships. Longitudinal studies are required to further confirm these findings. (2) High BP should be established reliably based on multiple measurements on 24-h ambulatory or home recordings. Thus, the reproducibility might not be ideal and there might be an overestimation of the prevalence in our study. (3) Due to the relative large sample size, we have not analyzed microalbuminuria and only used eGFR as criteria for classifying CKD. (4) Hormone levels were not measured in our study, and the mechanism could not be further studied.

## Conclusion

In summary, mildly decreased eGFR was significantly associated with concentric remodeling and concentric LVH, and moderate-severely decreased eGFR was an independent and significant risk factor for concentric LVH in men. In rural areas of Northeast China, the impaired eGFR and CVD are still major public health problems and highly prevalent. The public and government should pay more attention to renal function examination in rural areas in China. Future analysis of the rural population will clarify whether the incidence of LVH varies with progression of renal dysfunction during further follow-up.

## Abbreviations

BP: Blood pressure
CI: Confidence interval
CKD: Chronic kidney disease
CVD: Cardiovascular disease
DBP: Diastolic BP
EDTA: Ethylenediaminetetraacetic acid
eGFR: Estimated glomerular filtration rate
FPG: Fasting plasma glucose
HDL-C: High density lipoprotein-cholesterol
IVST: End-diastolic interventricular septum thickness
LDL-C: Low density lipoprotein-cholesterol
LVH: Left ventricular hypertrophy
LVM: Left ventricular mass
LVMI: Left ventricular mass index
OR: Odds ratio
PWT: End-diastolic posterior wall thickness
RWT: Myocardial relative wall thickness
SBP: Systolic BP
TC: Total cholesterol
TG: Triglyceride
